# CD8^+^ Regulatory T Cell Deficiency in Elderly-Onset Rheumatoid Arthritis

**DOI:** 10.3390/jcm12062342

**Published:** 2023-03-17

**Authors:** Ryu Watanabe, Keiichiro Kadoba, Atsuko Tamamoto, Koichi Murata, Kosaku Murakami, Hideo Onizawa, Takayuki Fujii, Akira Onishi, Masao Tanaka, Hiromu Ito, Akio Morinobu, Motomu Hashimoto

**Affiliations:** 1 Department of Clinical Immunology, Osaka Metropolitan University Graduate School of Medicine, Osaka 545-8585, Japan; 2 Department of Rheumatology and Clinical Immunology, Graduate School of Medicine, Kyoto University, Kyoto 606-8507, Japan; 3 Department of Advanced Medicine for Rheumatic Diseases, Graduate School of Medicine, Kyoto University, Kyoto 606-8507, Japan; 4 Department of Orthopaedic Surgery, Graduate School of Medicine, Kyoto University, Kyoto 606-8507, Japan; 5 Center for Cancer Immunotherapy and Immunobiology, Division of Clinical Immunology and Cancer Immunotherapy, Graduate School of Medicine, Kyoto University, Kyoto 606-8501, Japan; 6 Department of Orthopaedic Surgery, Kurashiki Central Hospital, Kurashiki 710-8602, Japan

**Keywords:** CD8, elderly-onset, matrix metalloprotease-3, regulatory T cell, rheumatoid arthritis

## Abstract

Elderly-onset rheumatoid arthritis (EORA) is associated with higher disease activity and accelerated joint destruction compared with young-onset RA (YORA). However, the underlying immunological mechanism remains unclear. Regulatory T cells (Tregs) are an immunosuppressive T cell subset, and CD4^+^ Tregs are deficient and/or dysfunctional in RA; however, CD8^+^ Tregs have not been fully examined in RA. Here, we aimed to determine the role of CD8^+^ Tregs, particularly in EORA. A total of 40 patients (EORA, *n* = 17; YORA, *n* = 23) were cross-sectionally enrolled. Current disease activity and treatment were comparable between the two groups; however, levels of multiple cytokines, including IL-1β, TNFα, interferon (IFN)-γ, IL-2, and IL-10, were significantly increased in EORA. The number of CD4^+^ Tregs did not differ between the groups (*p* = 0.37), but those of CD8^+^ Tregs were significantly decreased in EORA (*p* = 0.0033). The number of CD8^+^ Tregs were inversely correlated with plasma matrix metalloprotease (MMP)-3 levels (r = −0.3331, *p* = 0.036). Our study results revealed an intrinsic deficiency of CD8^+^ Tregs in patients with EORA, which leaves synovitis unchecked with excessive MMP-3 release. A therapeutic approach to restore CD8^+^ Tregs may provide a new avenue for the treatment of EORA.

## 1. Introduction

Rheumatoid arthritis (RA) is a chronic inflammatory disease that causes progressive joint destruction if not appropriately treated [1]. However, recent advances in treatment, particularly the advent of biological disease-modifying antirheumatic drugs (bDMARDs) and targeted synthetic DMARDs (tsDMARDs), have made it a feasible therapeutic goal to control joint destruction [2]. RA is prevalent among women aged in their 40s to 50s. However, with the aging of the population, the number of patients who develop RA after the age of 60 years has been increasing [3]. Compared with young-onset RA (YORA), elderly-onset RA (EORA) has a higher proportion of males, and many are seronegative for rheumatoid factor (RF) and anti-cyclic citrullinated peptide antibody (ACPA), which causes difficulties in differentiating EORA from polymyalgia rheumatica (PMR) [4,5]. Acute phase reactants, such as C-reactive protein (CRP) and erythrocyte sedimentation rate (ESR), are often elevated due to increased IL-6 signaling, which is associated with higher disease activity and more rapid joint destruction in EORA than in YORA [6,7]. However, the immunological mechanism underlying the difference between EORA and YORA remains largely unclear.

Regulatory T cells (Tregs) are a subset of CD4^+^ T cells that actively engage in immunological tolerance and prevent autoimmunity [8]. These cells are characterized by the expression of CD25 and the master transcription factor, Foxp3 [9]. Multiple studies have demonstrated that CD4^+^ Tregs are deficient and/or dysfunctional in autoimmune diseases, including RA [10,11,12], systemic lupus erythematosus (SLE) [13,14,15], giant cell arteritis (GCA) [16,17,18], PMR [19], and others.

Although CD4^+^ Tregs are well recognized, CD8^+^ Tregs are still controversial in many aspects, including their phenotypes and suppressive mechanisms [20]. However, accumulating evidence indicates that CD8^+^ Tregs also possess immunosuppressive functions, as found in animal models of inflammatory bowel disease, graft-versus-host disease, and viral infections [21,22,23]. In addition, although the number of CD8^+^ Tregs are not reduced in the elderly and patients with GCA, they are dysfunctional compared with those in younger individuals [24]; however, the role of CD8^+^ Tregs in EORA remains unknown.

Here, we aimed to determine whether plasma protein levels and the number of CD8^+^ and CD4^+^ Tregs differ between patients with EORA and YORA. We then assessed correlations between the number of CD8^+^ Tregs and plasma protein levels and examined the role of CD8^+^ Tregs in the pathophysiology of EORA.

## 2. Materials and Methods

### 2.1. Study Design and Selection of Patients

All patients who fulfilled the 1987 or 2010 classification criteria for RA [25,26] at Kyoto University Hospital were registered in the KURAMA cohort database, as previously described [27,28]. Patients with a diagnosis of RA were eligible for enrollment regardless of treatment and no exclusion criteria were set. Clinical data were recorded at baseline and at every visit. Patients who visited Kyoto University Hospital between April 2020 and March 2021 and had been treated with a treat-to-target strategy [29] were cross-sectionally enrolled. We defined YORA and EORA as RA with age at onset <60 and ≥60 years, respectively [6,7].

### 2.2. Clinical Evaluation

The medical records of the enrolled patients were retrospectively reviewed, as well as clinical data, including age, sex, disease duration, medication, erythrocyte sedimentation rate (ESR), serum C-reactive protein (CRP) values, swollen joint counts, tender joint counts, and titers of RF and ACPA. Rheumatoid factor and ACPA were considered positive if titers were >15 IU/mL and >4.5 U/mL, respectively. Disease activity of RA was monitored using the Disease Activity Score (DAS)28-ESR, DAS28-CRP, simplified disease activity index (SDAI), and clinical disease activity index (CDAI).

### 2.3. Measurement of Plasma Protein Levels

Plasma protein levels of IL-1β, IL-6, TNFα, interferon-γ (IFN-γ), IL-17, IL-2, IL-10, and matrix metalloproteinase (MMP)-3 were evaluated using the Luminex^®^ Discovery Assay Human Premixed Multi-Analyte Kit (Cat No. LXSAHM-20; R&D Systems Inc., Minneapolis, MN, USA) according to the protocol provided by the manufacturer.

### 2.4. Flow Cytometry

Peripheral blood mononuclear cells (PBMCs) were isolated from peripheral blood by density gradient centrifugation using BD Vacutainer^®^ CPT (Catalog No. 362753, BD Biosciences, San Jose, CA, USA). Flow cytometric analysis was performed using a BD LSRFortessa (BD Biosciences), and data were analyzed using FlowJo software (Tree Star, Ashland, OR, USA). Methods for measuring surface and intracellular proteins have previously been described [30]. APC-conjugated anti-human CD4 antibody (Clone A161A1, Catalog No. 357408), PerCP-conjugated anti-human CD8 antibody (Clone SK1, Catalog No. 344708), and FITC-conjugated anti-human Foxp3 antibody (Clone 206D, Catalog No. 320106) were obtained from BioLegend (San Diego, CA, USA). We used eBioscienceTM Foxp3/Transcription Factor Staining Buffer Set (Catalog No. 00-5523-00, ThermoFisher Scientific Inc., Waltham, MA, USA) to stain Foxp3. We defined CD4^+^Foxp3^+^ cells as CD4^+^ Tregs, and CD8^+^Foxp3^+^ cells as CD8^+^ Tregs.

### 2.5. Statistical Analysis

All statistical analyses were performed using Prism 9 (GraphPad Software Inc., La Jolla, CA, USA). The normality of all data was evaluated using Kolmogorov–Smirnov tests. Statistical significance was determined using unpaired two-tailed Student *t*-tests for normally distributed data, and Mann–Whitney U tests for data that were not normally distributed. Correlations were determined using Pearson or Spearman analyses based on the data distribution. Categorical variables were analysed using Fisher’s exact test. The Benjamini–Hochberg step-down procedure was applied to adjust for multiple tests and to control the false-discovery rate at 0.05 [31]. Values with *p* < 0.05 were considered significant.

### 2.6. Ethics Approval and Consent to Participate

The study protocol was approved by the Kyoto University Ethics Committee (R0357). All participants provided written informed consent to all study procedures, which complied with the principles of the Declaration of Helsinki.

## 3. Results

### 3.1. Clinical Characteristics of Enrolled Patients

Table 1 summarizes the clinical characteristics of the 40 patients (EORA, *n* = 17; YORA, *n* = 23) included in this study. In line with previous studies [4,5], our patients with EORA were significantly older (*p* < 0.001), included fewer females (*p* = 0.023), and had lower positivity for RF (*p* = 0.003) and ACPA (*p* < 0.001) compared with patients with YORA. Current disease activity and treatment including methotrexate, prednisolone, and biologics were balanced (Table 1). None of the patients were administered with Janus kinase (JAK) inhibitors.

### 3.2. Inflammatory Milieu Persisted despite Treatment in EORA

**Figure 1:**
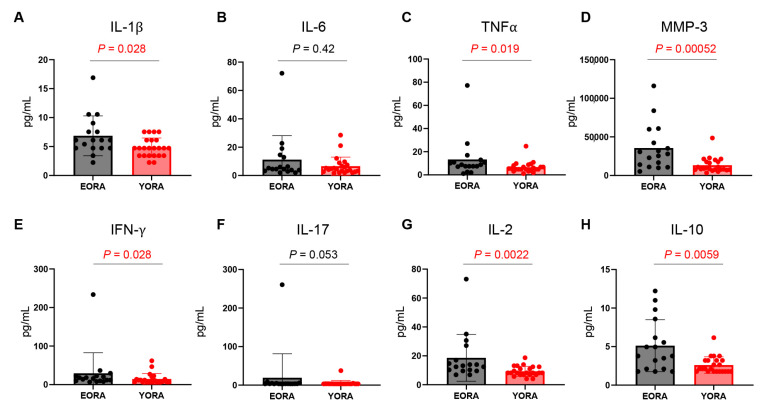
Comparison of plasma protein levels between elderly-onset rheumatoid arthritis (EORA) and young-onset RA (YORA). Plasma protein levels were compared between EORA (*n* = 17) and YORA (*n* = 23) groups. Data are shown as means ± SD. Statistical significance was calculated using unpaired two-tailed Student t-tests or Mann–Whitney U tests. (**A**) IL-1β, (**B**) IL-6, (**C**) tumor necrosis factor (TNF) α, (**D**) matrix metalloprotease (MMP)-3, (**E**) interferon (IFN)-γ, (**F**) IL-17, (**G**) IL-2, and (**H**) IL-10.

We measured the levels of plasma protein in the patients (Figure 1). Despite similar disease activity, levels of IL-1β (*p* = 0.028, Figure 1a), TNFα (*p* = 0.019, Figure 1c), MMP-3 (*p* = 0.00052, Figure 1d), IFN-γ (*p* = 0.028, Figure 1e), IL-2 (*p* = 0.0022, Figure 1g), and IL-10 (*p* = 0.0059, Figure 1h) were significantly increased in patients with EORA compared with YORA. Even after the multiple tests using the Benjamini–Hochberg procedure, the differences remained statistically significant. In contrast, IL-6 (*p* = 0.42, Figure 1b) and IL-17 (*p* = 0.053, Figure 1f) levels did not differ between the groups. These results indicated that the pathophysiology of EORA and YORA may fundamentally differ and that current therapies can suppress IL-6 and disease activity but cannot sufficiently diminish the inflammatory milieu.

### 3.3. CD8^+^ Tregs Are Deficient in EORA

**Figure 2:**
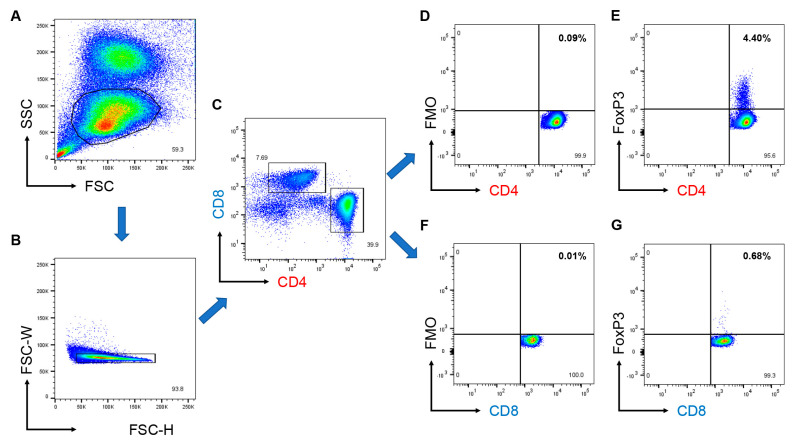
Gating strategy to identify CD4^+^ and CD8^+^ regulatory T cells (Tregs) using flow cytometry. (**A**) Lymphocytes from peripheral blood mononuclear cells gated using forward (FSC) and side (SSC) scatter. (**B**) Doublets were removed. (**C**) Identification of CD4^+^ and CD8^+^ T cells. (**D**) CD4^+^ fluorescent minus one (FMO), (**E**) CD4^+^Foxp3^+^ cells defined as CD4^+^ Tregs. (**F**) CD8^+^ FMO, (**G**) CD8^+^Foxp3^+^ cells defined as CD8^+^ Tregs.

**Figure 3:**
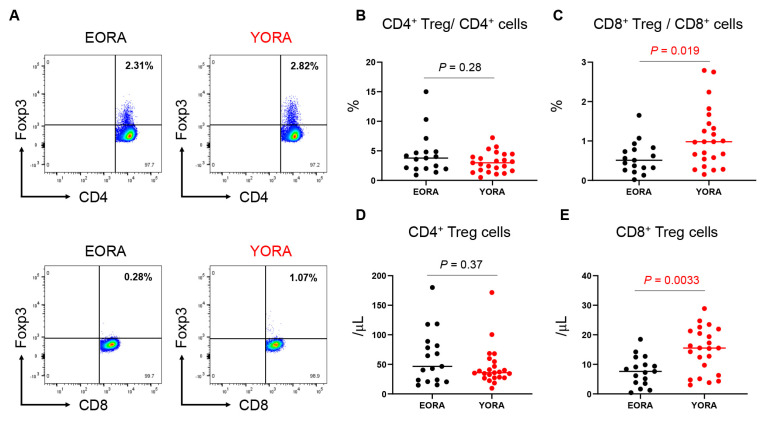
CD8^+^ regulatory T cells (Tregs) are deficient in elderly-onset rheumatoid arthritis (EORA). Number of CD4^+^ and CD8^+^ Tregs, and proportions of CD4^+^ and CD8^+^ Tregs to CD4^+^ and CD8^+^ T cells, were determined using flow cytometry (Figure 2 shows gating strategy). (**A**) Representative dot plots of CD4^+^ and CD8^+^ Tregs in patients with EORA and YORA. Proportions (%) of (**B**) CD4^+^ Tregs to CD4^+^ T cells and (**C**) CD8^+^ Tregs to CD8^+^ T cells. Comparison of absolute number of (**D**) CD4^+^ Tregs and (**E**) CD8^+^ Tregs in peripheral blood (/μL) between EORA (*n* = 17) and YORA (*n* = 23). (**B**–**E**) Data are shown as dot plots and medians, and statistical significance was determined using unpaired two-tailed Student *t*-tests or Mann–Whitney U tests. EORA, elderly-onset rheumatoid arthritis; YORA, young-onset rheumatoid arthritis.

We then measured the number of CD4^+^ and CD8^+^ Tregs, as well as the proportion of the cells (CD4^+^ Tregs and CD8^+^ Tregs to CD4^+^ and CD8^+^ T cells, respectively) using flow cytometry (Figure 2 and Figure 3). The gating strategy is shown in Figure 2. The proportion of CD4^+^ Tregs mostly ranged from 1%–5% (Figure 3b), whereas that of CD8^+^ Tregs ranged from 0%–2% (Figure 3c). When we compared these cells between patients with EORA and YORA (Figure 3a), the proportions and the number of CD4^+^ Tregs did not differ between the groups (Figure 3b,d), whereas those of CD8^+^ Tregs were significantly decreased in EORA (Figure 3c,e, *p* = 0.019, *p* = 0.0033, respectively). These differences remained statistically significant after multiple tests. Although a previous study demonstrated that CD8^+^ Tregs were comparable between young (<30 years) and elderly (>60 years) individuals [24], our results showed that CD8^+^ Tregs, but not CD4^+^ Tregs, are decreased in EORA.

### 3.4. Number of CD8^+^ Tregs Are Associated with Plasma MMP-3 Levels

**Figure 4:**
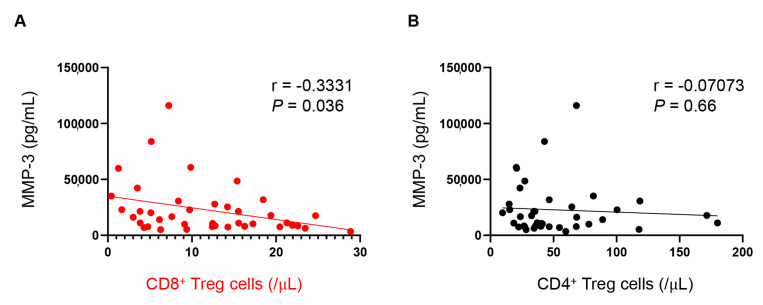
Inverse correlation between number of CD8^+^, but not CD4^+^ regulatory T cells (Tregs), with plasma matrix metalloprotease (MMP)-3 levels. Associations between number of CD8^+^ Tregs (**A**) or CD4^+^ Tregs (**B**) and plasma MMP-3 levels were examined using Pearson correlation analysis (*n* = 40).

Multiple cytokines were increased, whereas CD8^+^ Tregs were decreased in EORA. We then examined correlations between the abundance of CD8^+^ Tregs, age, RA disease activity, and plasma protein levels to determine whether the decrease in CD8^+^ Tregs is associated with the disease state of EORA (Table 2). Age (r = −0.2847, *p* = 0.075) and RA disease activity (DAS28-ESR, DAS28-CRP, SDAI, and CDAI, *p* = 0.61, *p* = 0.87, *p* = 0.93, and *p* = 0.89, respectively) were not associated with the abundance of CD8^+^ Tregs. Sex and seropositivity were also not associated with the number of CD8^+^ Tregs (*p* = 0.11, *p* = 0.23, respectively). Among plasma proteins, levels of MMP-3 were inversely correlated with the abundance of CD8^+^ Tregs (r = −0.3331, *p* = 0.036, Figure 4a), but not CD4^+^ Tregs (r = −0.07073, *p* = 0.66, Figure 4b). These results suggest that CD8^+^ Tregs may play a protective role in suppressing synovitis, particularly in elderly persons.

## 4. Discussion

We showed that an array of inflammatory cytokines persisted in EORA despite treatment. We also found that CD8^+^ Tregs, but not CD4^+^ Tregs, were deficient in patients with EORA, and that the number of CD8^+^ Tregs was inversely correlated with plasma MMP-3 levels. These results suggest that patients with EORA may have an intrinsic deficiency of CD8^+^ Tregs, which leaves synovitis unchecked, leading to excess release of MMP-3. Thus, restoring CD8^+^ Tregs may offer a new avenue for treating EORA.

In our study, levels of CRP, ESR, and IL-6 did not differ between patients with EORA and YORA, whereas those of IL-1β, TNFα, IFN-γ, and IL-2 were increased in EORA (Figure 1). Since synovial fibroblasts are the major producers of IL-6 [32], these results suggest that IL-6 production in synovial fibroblasts could be susceptible to treatment. In contrast, monocytes/macrophages and T cells, which are the producers of IL-1β, TNFα, and IFN-γ, may be persistently activated despite treatment in EORA. IL-2 is primarily produced by activated CD4^+^ T cells and functions as a major growth factor for CD4^+^ Tregs [33], but they were not increased in EORA. IL-10 can be secreted by not only CD4^+^ and CD8^+^ Tregs [33,34], but also Th1, Th2, B, and dendritic cells [35,36], which explains why IL-10 accumulated in EORA. These results suggest that the treatment that EORA patients were receiving failed to fundamentally correct the pathology of EORA.

In our recent work, we showed that IFN-γ is associated with the treatment resistance to anti-TNF inhibitor therapy [37]. In this multi-omics analysis involving 27 bDMARD-naïve RA patients, we found that, compared to responders, IFN-γ is accumulated during anti-TNF therapy in non-responders, which attracts additional T cells into the synovial tissue via CXC motif chemokine ligand 10, forming a vicious cycle of resistance to anti-TNF inhibitors [37]. Since IFN-γ utilizes the JAK-signal transducer and activator of transcription (STAT) pathway for intracellular signaling, our findings thus provide a rationale for the use of JAK inhibitors against EORA, although prior risk stratification is required [38].

Although the number of CD8^+^ Tregs do not decrease, their suppressive function is not maintained with age [24]. However, the potential to induce CD8^+^ Tregs from PBMCs using IL-15 and low-dose anti-CD3 is impaired in elderly compared with younger individuals [39]. We found that age did not correlate with the number of CD8^+^ Tregs, although the abundance was distinctly lower in patients with EORA than YORA (Figure 3). The decrease in CD8^+^ Tregs has been reported in other autoimmune diseases, such as SLE [40] and type 1 diabetes mellitus [41]. Our study results are consistent with these studies.

The suppressive activity of CD8^+^ Tregs is not mediated by IL-10 but relies on interference with the T cell receptor (TCR)-induced signaling cascade [39]. Specifically, CD8^+^ Tregs release exosomes containing NADPH oxidase 2 (NOX2) that interfere with the TCR-induced phosphorylation of ZAP-70 and suppress activation in neighboring CD4^+^ T cells [42]. Defective CD8^+^ Treg functions in elderly individuals and patients with GCA are attributed to the inadequate release of exosomes containing NOX2 [24]. We did not analyze the functions of CD8^+^ Tregs because they comprise a distinctly low proportion of T cells, particularly in EORA. Further studies are needed to determine the functional activity of CD8^+^ Tregs in patients with EORA.

Recently, low-dose IL-2 therapy has been expected to be effective against autoimmune diseases such as SLE because it can expand CD4^+^ Tregs [33,43]. The anti-IL-6 inhibitor tocilizumab can also restore the number and functions of CD4^+^ Tregs [17,44,45]; however, these effects on CD8^+^ Tregs are unknown [46]. Several attempts to expand CD8^+^ Tregs using probiotics or by in vitro or in vivo procedures are under investigation to treat autoimmunity [20,47].

In the present study, anti-IL-6 inhibitors, including tocilizumab and sarilumab, were used in six patients with YORA (26.1%) and only one with EORA (5.9%) (*p* = 0.21). The use of anti-IL-6 inhibitors did not affect the number of both CD4^+^ and CD8^+^ Tregs in this study (*p* = 0.68 and *p* = 0.78, respectively, Supplementary Figure S1). In addition, abatacept, a selective inhibitor for T cell activation, was administered in three cases of both YORA and EORA (*p* = 1.0). The use of abatacept also did not have an impact on the number of both CD4^+^ and CD8^+^ Tregs (*p* = 0.81 and *p* = 0.29, respectively, Supplementary Figure S1). Therefore, the use of these biological agents may not explain CD8^+^ Tregs’ deficiency in EORA in this study. 

The number of CD8^+^ Tregs was inversely correlated with plasma MMP-3 levels (Figure 4), but not RA disease activity and other cytokines (Table 2). The direct evidence showing that the deficiency of CD8^+^ Tregs causes excessive release of MMP-3 is scarce even in the literature, thus it remains unclear why MMP-3 is specifically associated with the number of CD8^+^ Tregs. Whether this is just a coincidence or not requires further investigation in the future.

The present study had several limitations. First, we included only 40 patients, and their clinical characteristics of EORA and YORA significantly differed (Table 1). The differences in age, sex, and seropositivity between EORA and YORA may have affected the number of CD8^+^ Tregs. However, the patient profiles were relatively typical of YORA or EORA and disease activity was comparable between the groups. Second, this study did not compare the results with those of healthy controls. Patients with EORA should be compared with age-matched, healthy, elderly individuals. Third, because of the cross-sectional study design, many patients had already been treated, which may have modified the results. The number of CD8^+^ Tregs should have been examined before and after treatment. Fourth, the definition of CD8^+^ Treg was not rigorous. Since the definition of the cell differs among reports [20,48], we defined CD8^+^Foxp3^+^ cells as CD8^+^ Tregs. Finally, as described above, the present study did not perform the functional assay of CD8^+^ Tregs.

## 5. Conclusions

We revealed that the number of CD8^+^ Tregs decreased and was inversely correlated with plasma MMP-3 levels in EORA. Further studies are required to utilize these cells for EORA treatment.

## Figures and Tables

**Table 1:** Comparison of clinical characteristics between elderly-onset and young-onset rheumatoid arthritis.

		EORA	YORA	*p*-Value
N		17	23	
Current age (y)		75 [71, 79]	61 [51, 65]	<0.001
Female (*n*, %)		10 (58.8%)	21 (91.3%)	0.023
Disease duration (mo)		106 [80, 220]	93 [38, 197]	0.23
RF-positive (*n*, %)		8 (47.1%)	21 (91.3%)	0.003
RF titers		9.3 [8.0, 32.6]	54.7 [26.1, 112.6]	0.003
ACPA-positive (*n*, %)		5 (29.4%)	21 (91.3%)	<0.001
ACPA titers		0.6 [0.5, 12.9]	91.6 [21.1, 164.5]	0.002
CRP (mg/dL)		0.10 [0.10, 0.20]	0.10 [0.10, 0.10]	0.33
ESR (mm/h)		17.0 [11.0, 43.0]	14.0 [7.0, 29.5]	0.36
DAS28-CRP		1.46 [1.28, 2.23]	1.52 [1.36, 2.37]	0.40
DAS28-ESR		2.72 [1.98, 3.10]	2.58 [1.94, 3.63]	0.61
SDAI		1.90 [0.90, 5.10]	1.90 [1.15, 6.50]	0.38
CDAI		1.30 [0.70, 3.60]	1.80 [1.05, 6.35]	0.20
MTX use (*n*, %)		11 (64.7%)	18 (78.3%)	0.48
PSL use (*n*, %)		7 (41.2%)	6 (26.1%)	0.50
MTX dose (mg/week)		6.0 [0, 10.0]	4.0 [0, 8.0]	0.28
PSL dose (mg/day)		0 [0, 1.0]	0 [0, 4.25]	0.19
Biologics use (*n*, %)		9 (52.9%)	15 (65.2%)	0.52
	IFX	1 (5.9%)	2 (8.7%)	1
	ADA	0 (0.0%)	2 (8.7%)	0.50
	ETN	0 (0.0%)	1 (4.3%)	1
	GLM	4 (23.5%)	1 (4.3%)	0.14
	TCZ	1 (5.9%)	5 (21.7%)	0.22
	SAR	0 (0.0%)	1 (4.3%)	1
	ABT	3 (17.6%)	3 (13.0%)	1
First Biologics (*n*, %)		4 (23.5%)	10 (43.5%)	0.32

Data are shown as median [interquartile range] for continuous variables and as numbers (%) for categorical variables unless otherwise stated. Continuous variables were analyzed using unpaired two-tailed Student *t*-test or Mann–Whitney U test, as appropriate. Categorical data were analyzed using Fisher’s exact test. ABT, abatacept; ACPA, anti-cyclic citrullinated peptide antibodies; ADA, adalimumab; CDAI: clinical disease activity index; CRP, C-reactive protein; DAS28: Disease Activity Score 28-joint count; EORA, elderly-onset rheumatoid arthritis; ESR: erythrocyte sedimentation rate; ETN, etanercept; GLM, golimumab; IFX, infliximab; MTX, methotrexate; PSL, prednisolone; RF: rheumatoid factor; SAR, sarilumab; SDAI: simplified disease activity index; TCZ, tocilizumab; YORA, young-onset rheumatoid arthritis.

**Table 2:** Correlations between number of CD8^+^ regulatory T cells and other variables.

	Pearson r	95% CI	*p* Value
Age	−0.2847	−0.5476 to 0.02942	0.075
DAS28-ESR	−0.08311	−0.3847 to 0.2345	0.61
DAS28−CRP	−0.0261	−0.3349 to 0.2877	0.87
SDAI	0.01433	−0.2985 to 0.3244	0.93
CDAI	0.02271	−0.2909 to 0.3319	0.89
IL-1β	−0.2481	−0.5195 to 0.06872	0.12
IL-6	−0.1732	−0.4599 to 0.1462	0.29
TNFα	−0.01798	−0.3277 to 0.2952	0.91
MMP-3	−0.3331	−0.5840 to −0.02404	0.036
IFN-γ	−0.1332	−0.4270 to 0.1861	0.41
IL-17	−0.04573	−0.3522 to 0.2696	0.78
IL-2	−0.2363	−0.5103 to 0.08118	0.14
IL-10	−0.2569	−0.5263 to 0.05937	0.11

Pearson correlation analysis. CDAI: clinical disease activity index; CI: confidence interval; CRP, C-reactive protein; DAS28: Disease Activity Score 28-joint count; ESR: erythrocyte sedimentation rate; IFN: interferon; IL: interleukin; MMP; matrix metalloprotease; SDAI: simplified disease activity index; TNF: tumor necrosis factor.

## Data Availability

Not applicable.

## Supplementary Materials

The following supporting information can be downloaded at: https://www.mdpi.com/article/10.3390/jcm12062342/s1, Figure S1: Numbers of CD4^+^ and CD8^+^ regulatory T cells (Tregs) sorted by the use of anti-IL-6 inhibitors and abatacept.
