# Supplementary material for: CD8+ Regulatory T Cell Deficiency in Elderly-Onset Rheumatoid Arthritis
Source: J Clin Med. 2023 Mar 17;12(6):2342. doi: 10.3390/jcm12062342 (PMC10054757; doi:10.3390/jcm12062342)
Supplement: Supplementary file 1 [file jcm-12-02342-s001.zip › jcm-2225091-supplementary.pdf]

CD8+ regulatory T cell deficiency in elderly-onset rheumatoid arthritis

Supplementary

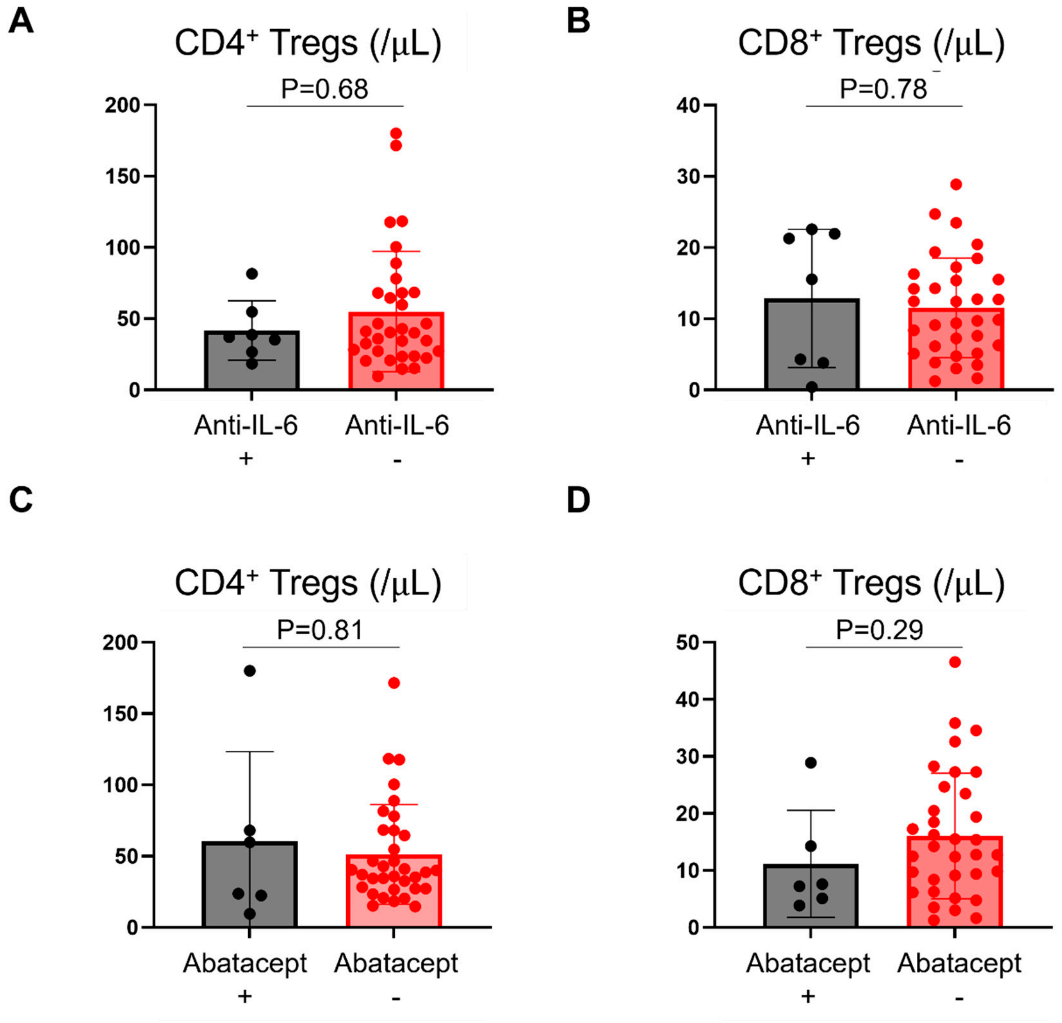

Supplementary Figure S1. Numbers of CD4+ and CD8+ regulatory T cells (Tregs) sorted by the use of anti-IL-6 inhibitors and abatacept.

Numbers of (A) CD4+ Tregs (/μL) and (B) CD8+ Tregs (/μL) sorted by the use of anti-IL-6 inhibitors. Numbers of (C) CD4+ Tregs (/μL) and (D) CD8+ Tregs (/μL) sorted by the use of abatacept. Mann-Whitney test.
